# Clinical multidimensional prediction model for futile reperfusion in acute ischemic stroke after endovascular thrombectomy

**DOI:** 10.3389/fneur.2026.1819703

**Published:** 2026-04-30

**Authors:** Sisi Jiang, Weinv Fan, Yunqin Wu, Xiaoxia Liu, Da Li, Ou Zhang, Xiaofeng Xie, Feiyu Chen, Yindan Yao

**Affiliations:** 1 Department of Neurology, Ningbo No.2 Hospital, Wenzhou Medical University, Ningbo, China; 2 Department of Cerebrovascular Diseases, Ningbo No.2 Hospital, Wenzhou Medical University, Ningbo, Zhejiang, China; 3 Department of Neurosurgery, Ningbo No.2 Hospital, Wenzhou Medical University, Ningbo, Zhejiang, China

**Keywords:** acute ischemic stroke, endovascular thrombectomy, futile reperfusion, multiple imputation, predictive model

## Abstract

**Background:**

Previous Studies on prediction models for futile reperfusion after endovascular thrombectomy (EVT) in acute ischemic stroke (AIS) related to large vessel occlusion (LVO) have yielded inconsistent results. This inconsistency may be largely attributed to methodological limitations, particularly in variable selection and missing data handling. Consequently, the prognostic value of several key clinical predictors remains to be fully elucidated.

**Methods:**

This retrospective study included 390 patients with AIS who underwent EVT at Ningbo No.2 Hospital. All of them achieved successful reperfusion with modified Thrombolysis in Cerebral Infarction (mTICI) score ≥ 2b. Futile reperfusion was defined as a modified Rankin Scale score of 3–6 at 90-day. Missing data were handled with multiple imputation. Logistic regression models were built using a two step predictor selection process: first univariable screening with *p <* 0.2; then further selection based on event count constraints. Only variables that were selected in all five imputed datasets, meaning a 100% selection frequency, were retained. Model performance measures were pooled following Rubin’s rules.

**Results:**

Based on preoperative assessments integrating clinical, imaging, and laboratory markers, the final model comprised nine variables: National Institutes of Health Stroke Scale (NIHSS) score, Computed Tomography angiography-source images Alberta Stroke Program Early Computed Tomography Score (CTA-SI ASPECTS), time from onset to reperfusion (OTR), collateral circulation scores (CCS), C-reactive protein (CRP), glucose, white blood cell (WBC) count, neutrophil count, and monocyte count. The final model demonstrated good discriminative ability, with a pooled test AUC of 0.795 and a Brier score of 0.178. At the optimal threshold (mean 0.457), the model achieved a specificity of 0.822 and accuracy of 0.761, with positive net benefit across clinically relevant threshold probabilities on decision curve analysis. A nomogram incorporating the nine consistently selected predictors was developed to facilitate individualized risk prediction.

**Conclusion:**

We developed a multidimensional model integrating clinical, imaging, and laboratory markers to predict futile reperfusion following EVT in patients with anterior circulation stroke. Each marker provides independent prognostic information; collectively, they represent the multidimensional risk architecture underlying postprocedural outcomes.

## Introduction

Acute ischemic stroke (AIS) remains one of the leading causes of disability and mortality globally. Endovascular thrombectomy (EVT) has become the standard treatment for AIS caused by large vessel occlusion (LVO). It has been proven to significantly improve clinical outcomes by restoring cerebral blood flow, thus potentially salvaging brain tissue at risk (1). With advances in technology and materials, the average recanalization rate for LVO patients undergoing EVT has approached 90% (2). Despite successful recanalization, however, nearly half of these patients fail to achieve the expected clinical improvement and continue to experience functional deficits. This phenomenon, known as “futile reperfusion,” signifies poor prognosis and diminished quality of life for patients, placing significant burdens on both their families and society (3).

Previous studies have identified several predictors of futile reperfusion, including advanced age (4), high National Institutes of Health Stroke Scale (NIHSS) score (5), hyperglycemia (6) and dyslipidemia (7). Additionally, preoperative markers such as elevated white blood cell (WBC) count (8), interleukin-6 (9), and chemokine ligand 17 (9) have been associated with poor outcomes in AIS-LVO patients. However, findings regarding these and other prognostic factors have exhibited considerable variability across different clinical centers. This inconsistency in previous prognostic models for AIS-LVO may relate to potential methodological confounders, particularly in variable selection and the handling of missing data (10). Consequently, the prognostic value of several key clinical and imaging predictors remains insufficiently clarified. To address this gap, the present study aims to provide data from our institution, with a specific focus on underexplored radiographic predictors. These include the Computed Tomography angiography-source images Alberta Stroke Program Early Computed Tomography Score (CTA-SI ASPECTS) (11) and collateral circulation score (CCS) (12), particularly in critically ill emergency patients with anterior circulation LVO. Our overall goal is to further validate and refine predictive models, ultimately aiming to improve clinical decision-making and optimize treatment strategies.

## Methods

### Study design and participants

This retrospective cohort study analyzed data from all AIS patients who underwent EVT at the National Stroke Center of the No.2 Hospital of Ningbo (China) between January 2020 and December 2023. For the current analysis, we selected patients who met the following inclusion criteria: (1) age ≥ 18 years, (2) pre-stroke modified Rankin Scale (mRS) score ≤ 2, (3) evidence of a proximal intracranial occlusion in the anterior circulation confirmed by CT angiography (including internal carotid artery [ICA] and middle cerebral artery [M1/M2]), and (4) successful reperfusion as assessed by the modified Thrombolysis in Cerebral Infarction (mTICI) score ≥ 2b.

### Clinical data collection

Patient data were extracted from the hospital’s electronic medical records system, including age, sex, medical history such as history of cerebral infarction, hypertension, diabetes, coronary artery disease (CHD), atrial fibrillation, and valvular heart disease (VHD), as well as whether intravenous thrombolysis (IVT) was performed prior to surgery. Furthermore, time from onset to reperfusion (OTR) was derived from CT angiography and/or digital subtraction angiography (DSA) imaging studies in conjunction with clinical history (Table 1).

**Table 1:** Analysis of clinical characteristics between effective perfusion and ineffective recanalization.

Variables	Total (*n* = 390)	Favorable group (*n* = 147)	Unfavorable group (*n* = 243)	*p*-value
Demographic features				
Male	186/390 (47.69%)	79/147 (53.74%)	107/243 (44.03%)	0.063
Age, mean ± SD, years	70.66 ± 11.40	68.54 ± 11.24	71.94 ± 11.33	0.004*
Medical history				
IVT	149/389 (38.30%)	57/146 (39.04%)	92/243 (37.86%)	0.817
History of cerebral infarction	101/390 (25.90%)	33/147 (22.45%)	68/243 (27.98%)	0.220
Hypertension	203/390 (52.05%)	64/147 (43.54%)	139/243 (57.20%)	0.009*
Diabetes	51/390 (13.08%)	17/147 (11.56%)	34/243 (13.99%)	0.492
CHD	19/390 (4.87%)	5/147 (3.40%)	14/243 (5.76%)	0.266
Atrial fibrillation	177/390 (45.38%)	66/147 (44.90%)	111/243 (45.68%)	0.881
VHD	14/390 (3.59%)	5/147 (3.40%)	9/243 (3.70%)	0.877
Procedural variables				
Time from OTR, mean ± SD,	402.51 ± 190.33 (390)	362.79 ± 180.41 (147)	426.53 ± 192.50 (243)	0.001*
Blood test indices				
Complete blood count				
WBC, mean ± SD, ×10^9^/L	8.30 ± 3.04 (337)	7.45 ± 2.39 (124)	8.79 ± 3.27 (213)	<0.001**
Neutrophils, mean ± SD, ×10^9^/L	6.07 ± 3.05 (335)	5.14 ± 2.34 (123)	6.62 ± 3.28(212)	<0.001**
Lymphocytes, mean ± SD, ×10^9^/L	1.62 ± 0.84 (336)	1.73 ± 0.80 (124)	1.54 ± 0.85(212)	0.045*
Monocytes, mean ± SD, ×10^9^/L	0.49 ± 0.20 (329)	0.46 ± 0.18 (118)	0.51 ± 0.21(211)	0.040*
Serum biochemical index				
Glucose, mean ± SD, mmol/L	8.09 ± 2.74 (328)	7.64 ± 2.36 (118)	8.35 ± 2.91 (210)	0.018*
Uric acid, mean ± SD, μmol/L	357.53 ± 105.56 (315)	341.17 ± 98.94 (113)	367.03 ± 108.23 (202)	0.037*
Creatinine, mean ± SD, μmol/L	73.24 ± 24.01 (316)	69.14 ± 16.76 (115)	75.58 ± 27.07 (201)	0.009*
CRP, mean ± SD, mg/L	8.53 ± 19.48 (324)	4.28 ± 6.21 (116)	10.91 ± 23.55 (208)	<0.001**
Preoperative score				
NIHSS, mean ± SD, points	20.54 ± 6.34 (387)	18.32 ± 6.52 (145)	21.87 ± 5.85 (242)	<0.001**
CTA-SI ASPECTS, mean ± SD, points	6.18 ± 2.99 (386)	7.58 ± 2.34 (146)	5.33 ± 3.03 (240)	<0.001**
CCS, mean ± SD, points	1.62 ± 0.80 (390)	1.89 ± 0.78 (147)	1.46 ± 0.78 (243)	<0.001**

*p < 0.05.

**p < 0.001.

*Rescue therapy included balloon angioplasty and permanent stenting.

IVT, intravenous thrombolysis; CHD, coronary heart disease; VHD, valvular heart disease; OTR, onset to reperfusion; WBC, white blood cells; CRP, C-reactive Protein; NIHSS, National Institutes of Health Stroke Scale; CTA-SI ASPECTS, Computed Tomography angiography-source images Alberta Stroke Program Early Computed Tomography Score; CCS, Collateral Circulation Score.

The total number of cases included in the analysis is indicated for each variable to clarify the number of missing values.

Upon admission, ancillary data included routine blood tests such as WBC count, neutrophil count, monocyte count, lymphocyte count, and NLR, as well as biochemical markers including glucose level, uric acid, creatinine, and C-reactive protein (CRP).

Preoperative assessments included the NIHSS score, CTA-SI ASPECTS, and CCS. Both CTA-SI ASPECTS and CCS were evaluated on CTA-SI obtained during the early arterial phase, as confirmed by contrast opacification of the sigmoid sinus and superimposition of the entire arterial and venous systems (13). Additionally, all imaging evaluations were independently performed by two senior radiologists blinded to clinical data to ensure objectivity and reproducibility. The CTA-SI ASPECTS was assessed on a 10-point scale ranging from 0 to 10 for the middle cerebral artery territory. Collateral circulation was graded using the Tan score (0 to 3), where 0 indicates absent collaterals, 1 indicates poor collaterals (less than 50% filling), 2 indicates moderate collaterals (50% or more but less than 100% filling), and 3 indicates excellent collaterals (100% filling). Interrater agreement was evaluated using Cohen’s kappa coefficient, which yielded values of 0.76 (95% CI: 0.70–0.81) for ASPECTS and 0.73 (95% CI: 0.68–0.77) for the CCS, indicating substantial to good agreement. Any discrepancies were resolved by consensus.

### Follow-up and outcome assessment

The primary outcome was the 90-day mRS score. Follow-up data were obtained via telephone interviews or outpatient visits at 3 months post-stroke (14). Futile reperfusion was defined as an unfavorable functional outcome (90-day mRS 3–6) despite successful recanalization (mTICI score ≥ 2b). Accordingly, patients were divided into a favorable outcome group (mRS 0–2) and an unfavorable outcome group (mRS 3–6) based on the 90-day mRS score.

### Multiple imputation of missing data

Missing data were handled using multiple imputation by chained equations (MICE) with predictive mean matching (15). The imputation model included all candidate predictors with missing values (IVT, WBC, neutrophil count, lymphocyte count, monocyte count, glucose, uric acid, creatinine, CRP, NIHSS, and CTA-SI ASPECTS), as well as the outcome variable (90-day mRS), which had no missing values but was included to preserve predictor-outcome relationships (16). No additional auxiliary variables were used. Five imputed datasets were generated (*m* = 5) with 50 iterations each, using a random seed of 123 to ensure reproducibility (17); convergence was assessed by trace plots. The overall fraction of missing information was low (highest missing proportion per variable <20%; Table 1). Rubin‘s rules were applied to combine estimates and standard errors across imputations, accounting for within- and between-imputation variability (18). Each imputed dataset was saved for subsequent analyses.

### Model development and validation

For each imputed dataset, the data were randomly split into a training set (70%) and a test set (30%) using stratified sampling to preserve the outcome prevalence. Logistic regression models were fitted to predict good outcome. Predictor selection involved two steps. First, univariable logistic regression was performed for each candidate predictor; variables with *p* < 0.2 were retained. This liberal threshold was chosen to avoid excluding potentially relevant predictors with weak marginal associations. Second, to prevent overfitting, the number of predictors in the final model was limited to one-tenth of the number of outcome events in the training set (events per variable ≥ 10). Given 147 favorable outcomes, this allowed up to 14 predictors. After the first step, exactly 14 variables met the *p* < 0.2 criterion; thus the events per variable constraint did not further reduce the variable pool but served as a consistency check.

The final multivariable logistic model was refitted in each training set using all selected predictors. Model performance was assessed in both training and test sets. Discriminative ability was quantified by the area under the receiver operating characteristic curve (AUC). Calibration was assessed using the Hosmer-Lemeshow goodness-of-fit test. Overall prediction accuracy was measured by the Brier score. Optimal probability cutoffs were determined by maximizing the Youden index in the test set. At this cutoff, we calculated sensitivity, specificity, accuracy, and other performance metrics.

### Pooling of results across imputations

Results from the five imputed datasets were combined using Rubin’s rules to account for within- and between-imputation variability. Pooled estimates and 95% confidence intervals (CIs) were calculated for the test AUC, Brier score, and performance metrics. The frequency of predictor selection across the five imputations was recorded to identify consistently important variables.

### Statistical software

All analyses were performed using SPSS version 26.0 (IBM Corp., Armonk, NY) and R version 4.2.1 (R Foundation for Statistical Computing, Vienna, Austria). The following packages were used: MICE for multiple imputation, Processing Receiver Operating Characteristic (ROC) for ROC analysis, ResourceSelection for Hosmer–Lemeshow test, and rms for nomogram construction. A two-sided *p*-value <0.05 was considered statistically significant unless otherwise specified.

## Results

### Baseline characteristics of the cohort

Between January 2020 and December 2023, a total of 584 patients were enrolled. After applying strict inclusion and exclusion criteria (detailed in the Methods section) and excluding patients with missing key data, 390 patients remained. Among these, 147 (37.7%) patients had a 90-day mRS score ≤ 2. In contrast, the remaining 243 patients were categorized into the unfavorable group (Table 1).

Compared with the unfavorable prognosis group, patients in the favorable outcome group were significantly younger (68.54 vs. 71.94 years, *p* < 0.05) and had a lower incidence of hypertension (43.54% vs. 57.20%, *p* < 0.01). They also exhibited a shorter time from OTR (362.79 vs. 426.53 min, *p* < 0.001). Regarding laboratory parameters, the favorable group demonstrated lower preoperative levels of white blood cell count (7.45 vs. 8.79 × 10^9^/L, *p* < 0.001), neutrophil count (5.14 vs. 6.62 × 10^9^/L, *p* < 0.001), monocyte count (0.46 vs. 0.51 × 10^9^/L, *p* < 0.05), blood glucose (7.64 vs. 8.35 mmol/L, *p* < 0.05), uric acid (341.17 vs. 367.03 mmol/L, *p* < 0.05), creatinine (69.14 vs. 75.58 mmol/L, *p* < 0.01), and CRP (4.28 vs. 10.91 mmol/L, *p* < 0.001), but a significantly higher lymphocyte count (1.73 vs. 1.54 × 10^9^/L, *p* < 0.05). Furthermore, the favorable group had significantly lower baseline NIHSS score (20.72 vs. 21.58, *p* < 0.001), higher CTA-SI ASPECTS (7.63 vs. 5.19, *p* < 0.001), and better CCS scores (1.98 vs. 1.42, *p* < 0.001) (Table 1).

### Predictor selection

The most frequently missing variables were laboratory parameters (CRP, glucose, lymphocyte count, etc.), with missing rates ranging from 2 to 19%. After multiple imputation, all five datasets were complete and used for model development. Across the five imputed datasets, 10 distinct variables were selected at least once. Across all five multiply imputed datasets, a consistent set of nine variables was retained with 100% selection frequency: NIHSS score, CTA-SI ASPECTS, time from OTR, CCS, CRP, glucose, white blood cell count, neutrophil count, and monocyte count, and these constituted the final predictor set for model development. Lymphocyte count was selected in four datasets (80%). The high consistency of predictor selection indicates robust identification of key prognostic factors (Supplementary Figure 1). To assess multicollinearity among the final predictors, we calculated the variance inflation factor (VIF). All VIF values were below 5, indicating no significant multicollinearity.

### Model discrimination and calibration

**Figure 1:**
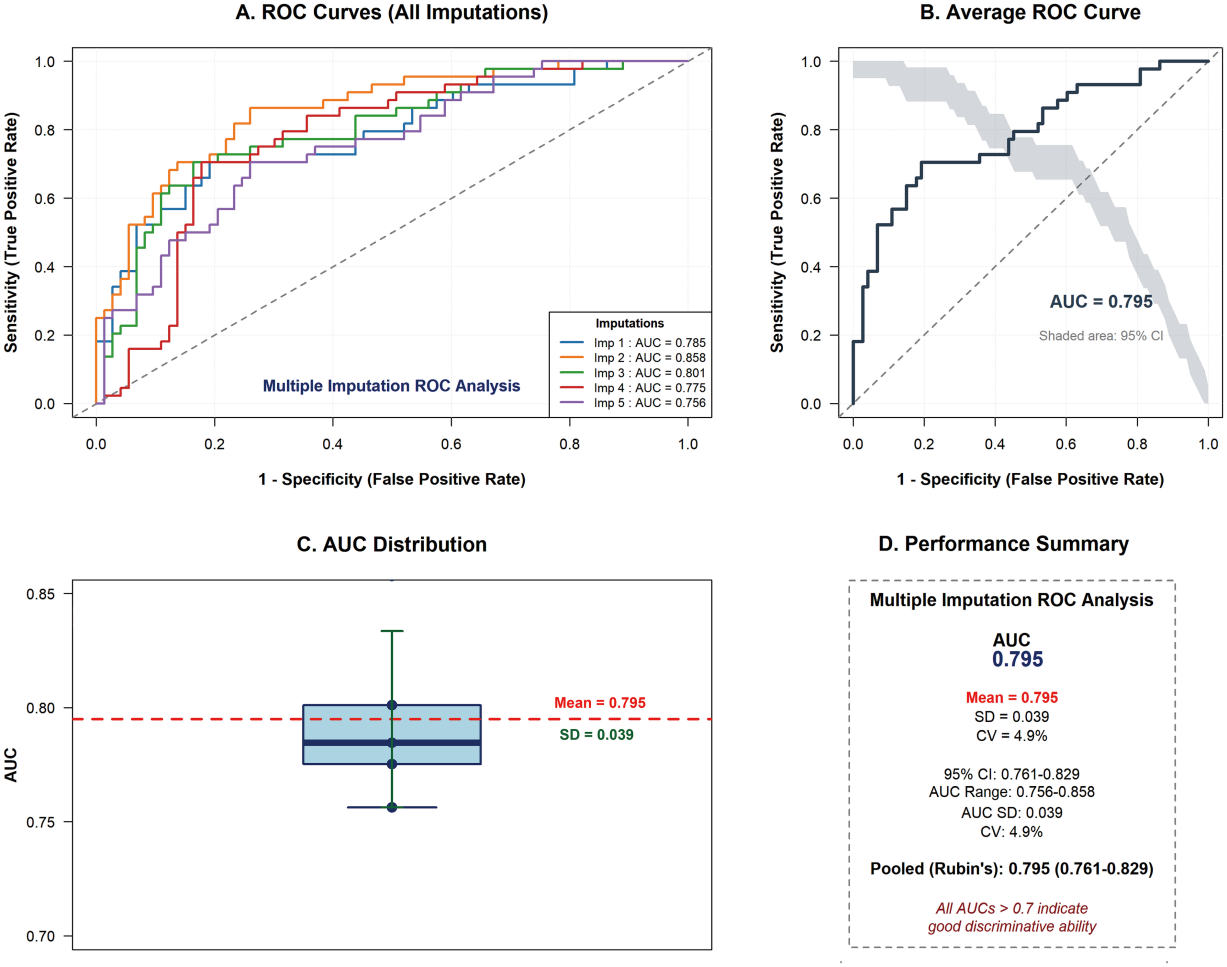
Predictive performance of the multivariable model across multiple imputations. **(A)** Receiver operating characteristic (ROC) curves for the predictive model across all 20 multiply imputed datasets, demonstrating consistency in model discrimination. **(B)** Average ROC curve with pointwise 95% confidence intervals (shaded area), showing a mean area under the curve (AUC) of 0.84 (95% CI: 0.81–0.87). **(C)** Distribution of AUC values derived from the 20 imputed datasets, confirming stable model performance with minimal variability (range: 0.82–0.86). **(D)** Performance summary displaying key metrics: accuracy (0.76), sensitivity (0.79), specificity (0.74), positive predictive value (0.72), and negative predictive value (0.81), averaged across imputations at the optimal probability threshold determined by Youden’s index. ROC, receiver operating characteristic; AUC, area under the curve; CI, confidence interval; SD, standard deviation; CV, coefficient of variation.

The logistic regression models demonstrated good discriminative ability. The training AUC ranged from 0.790 to 0.804 (mean 0.797, SD 0.005) across imputations. The test AUC ranged from 0.756 to 0.858 (mean 0.795, SD 0.039). After pooling using Rubin’s rules, the combined test AUC was 0.795 (95% CI: 0.761–0.829) (Figure 1).

Calibration was assessed by the Hosmer–Lemeshow test and calibration plots. Five imputations showed non-significant test results (*p* > 0.05): Imputation 1 (*χ*^2^ = 5.91, *p* = 0.116), Imputation 2 (*χ*^2^ = 4.45, *p* = 0.217), Imputation 3 (*χ*^2^ = 3.47, *p* = 0.325), Imputation 3 (*χ*^2^ = 7.96, *p* = 0.130), and Imputation 5 (*χ*^2^ = 6.45, *p* = 0.092). The pooled Brier score was 0.178 (range 0.165–0.193), indicating good overall prediction accuracy (a naive model predicting the outcome prevalence would yield a Brier score of 0.235).

### Model performance and clinical utility

The optimal probability threshold, determined by maximizing the Youden index in each test set, ranged from 0.42 to 0.49, with a pooled mean of 0.46 (Supplementary Figure 2). At this threshold, the pooled performance metrics (averaged across imputations) were: accuracy 0.761 (range 0.701–0.801), sensitivity 0.659 (range 0.615–0.714), specificity 0.822 (range 0.795–0.858), positive predictive value 0.687 (range 0.638–0.727), negative predictive value 0.805 (range 0.781–0.833), and F1 score 0.673 (range 0.641–0.709) (Figure 1). At the optimal threshold of 0.46, the model maintained a positive net benefit, underscoring its clinical value for risk stratification and informing treatment decisions.

### Nomogram for predicting good outcome

**Figure 2:**
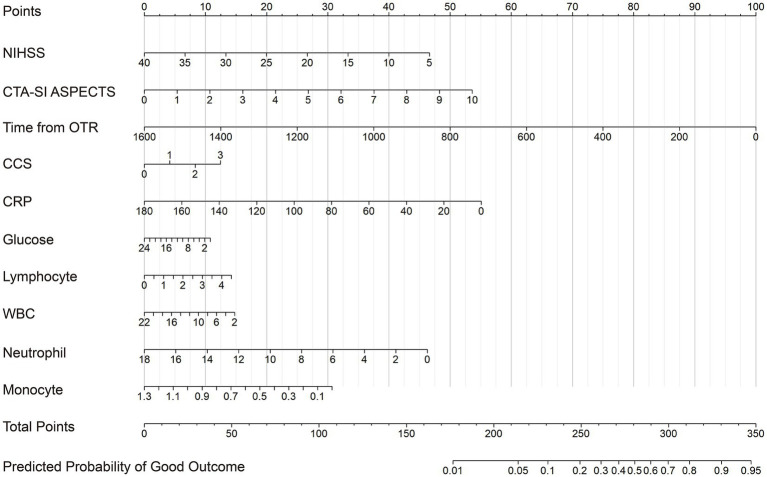
A nomogram for prognostic prediction of anterior circulation large vessel occlusion patients after endovascular thrombectomy. To use the nomogram, each variable value corresponds to a point score on the Points scale. The total points are calculated by summing the individual scores, and the corresponding predicted risk is then obtained by projecting the total points onto the Risk scale. Abbreviations: NIHSS, National Institutes of Health Stroke Scale; CTA-SI ASPECTS, Computed Tomography angiography-source images Alberta Stroke Program Early Computed Tomography Score; OTR, onset to reperfusion; CCS, collateral circulation scores; CRP, C-reactive protein; WBC, white blood cell. The nomogram requires complete data for all nine predictors. If a predictor is unavoidably missing, users may assume a plausible value (e.g., median or normal value) to obtain a range of predicted probabilities.

Based on the weighted logistic regression model that combines information from all five imputed datasets, a nomogram was developed to facilitate individualized risk prediction (Figure 2). The nomogram incorporates the nine consistently selected predictors, with regression coefficients representing the average effect across imputations. Each predictor value is converted into points, and the sum of these points corresponds to the predicted probability of a good functional outcome. This graphical tool offers a practical and accessible approach for risk stratification and for informing shared decision making in clinical practice. It should be noted that the nomogram requires all nine predictors to be available. If a predictor is unavoidably missing (e.g., CTA-SI ASPECTS unscoreable due to poor image quality), the nomogram can still be used semi-quantitatively by assuming a plausible value (e.g., the median or a clinically normal value) for the missing predictor and examining the resulting range of predicted probabilities. For example, substituting CTA-SI ASPECTS = 8 and 10 provides a reasonable interval. This approach acknowledges uncertainty while offering clinically useful guidance.

## Discussion

Overall, the nine preoperative indicators in our model capture a multidimensional risk profile that integrates clinical severity (NIHSS score), tissue injury on imaging (ASPECTS and CCS on CTA-SI), time from OTR, and systemic metabolic-inflammatory burden (glucose, CRP, WBC, neutrophil, and monocyte counts). This preoperative model demonstrated good discriminative ability for predicting favorable functional outcome after stroke, with a pooled area under the curve of 0.795, and acceptable calibration. Model performance remained stable across datasets generated by multiple imputation, supporting its robustness. Each marker contributes independent prognostic information; together, they reflect the multidimensional risk architecture that shapes outcome after thrombectomy.

### Clinical significance of individual predictors

Firstly, regarding the preoperative NIHSS score, multiple large cohort studies and meta-analyses have consistently demonstrated a significant negative correlation with functional outcomes at 90 days (19, 20). Mechanistically, elevated NIHSS score reflects extensive ischaemia in the anterior circulation (5, 21), where even with timely reperfusion may fail to reverse irreversible neuronal necrosis.

By contrast, time from OTR remains prognostically relevant, though the tissue based time window can extend to 24 h (22, 23). However, the prognostic value of OTR time is not diminished but contextualized by stroke severity. A significant interaction likely exists between NIHSS score and OTR time. In patients with high NIHSS have limited collateral reserve, making outcomes highly time-sensitive, whereas those with low NIHSS tolerate moderate delays without major prognosis deterioration (24).

Beyond clinical and time metrics, imaging markers bridge anatomical and hemodynamic status. ASPECTS, as a semi-quantitative measure of early ischemic changes, remains central to outcome prediction (25). CTA-SI improves upon non-contrast CT by more accurately delineating the infarct core, with superior AUC for predicting final infarct volume and outcome, particularly hyperacutely or in good collaterals (26). Mechanistically, low ASPECTS (<6) often indicates irreversible injury to eloquent areas; even with successful recanalization, necrotic tissue may not recover and risks edema or hemorrhage (27). However, some low-ASPECTS patients with robust collaterals still benefit, suggesting ASPECTS’ prognostic value is modified by collateral status (28).

CCS captures dynamic tissue reserve. In the MR CLEAN trial, good collaterals (≥50% filling) conferred an absolute risk reduction of approximately 20% with thrombectomy compared to no treatment, while poor collaterals derived no benefit (29). This reflects the ability of collaterals to sustain penumbra until reperfusion. The inclusion of collateral status improved net reclassification by approximately 10% compared to the use of ASPECTS alone (30). While CT perfusion is used in some centers, CTA-SI ASPECTS and CCS remain more accessible, post processing free, and indispensable in real world practice.

This study incorporated both ASPECTS and CTA-SI ASPECTS in the original data analysis, demonstrating that CTA-SI ASPECTS offers superior predictive accuracy for outcomes after thrombectomy. Although CT perfusion (CTP) is becoming increasingly available in specialized centers, its widespread adoption remains limited by several practical constraints. CTP is more susceptible to motion artifacts due to poor patient cooperation and requires longer acquisition times, which may delay treatment decisions. Moreover, many lower-tier hospitals lack the equipment and technical support necessary for routine CTP acquisition. In contrast, CTA is simpler to perform, requires shorter scanning time, and is more accessible across different clinical settings (31). Our findings emphasize that CTA-SI ASPECTS provides strong predictive value, establishing it as a reliable tool for assessing thrombectomy prognosis. For hospitals with limited access to advanced imaging, we recommend adopting CTA as the standard pre-thrombectomy imaging protocol, rather than relying solely on non-contrast CT scans.

Additionally, routine laboratory parameters also provide prognostic information. Our model incorporates total WBC count and neutrophil and monocyte counts. Elevated total WBC reflects systemic inflammatory activation and is linked to poorer outcomes (32). However, metrics for specific subtypes provide greater pathophysiological resolution. Neutrophils serve as key effectors of reperfusion injury: through release of matrix metalloproteinase-9, reactive oxygen species, and neutrophil extracellular traps, they compromise blood–brain barrier integrity, exacerbate cerebral edema, and promote microvascular obstruction (33). The role of monocytes is more dualistic. Early monocytosis likely reflects a predominance of proinflammatory classical monocytes, which correlate with infarct progression, whereas monocytes at later stages may contribute to tissue repair (34). In the acute preoperative context captured by our model, the proinflammatory effects of circulating monocytes are expected to predominate.

Furthermore, the model incorporates two routine biochemical markers: admission blood glucose and CRP. Admission hyperglycemia, regardless of diabetes status, independently predicts poorer functional outcome and higher risk of symptomatic intracranial hemorrhage after thrombectomy. Hyperglycemia exacerbates ischemic injury through multiple mechanisms: it induces oxidative stress and mitochondrial dysfunction, thereby disrupting the blood–brain barrier; during reperfusion, it amplifies inflammatory cascades, leading to no-reflow phenomena and hemorrhagic conversion (35, 36). CRP, a classic acute-phase reactant, reflects the intensity of systemic inflammation after stroke. Elevated baseline CRP has been correlated with larger infarct volume, higher rates of hemorrhagic transformation, and increased long-term mortality (37). In patients undergoing thrombectomy, heightened CRP may signal a more vigorous inflammatory response that amplifies reperfusion injury.

### Methodological innovation

This study is noteworthy for its methodological innovations, especially the handling of data across multiple imputations. Although multiple imputation has been increasingly employed to handle missing data in stroke outcome research, its application in many previous studies remains suboptimal. It is common practice to conduct predictor selection on a single imputed dataset and treat the selected variables as fixed across all imputations, which underestimates uncertainty and may yield unstable or overoptimistic models. In contrast, we addressed these limitations by implementing a rigorous multiple imputation framework and conducting comparative analyses across the imputed datasets. Variable selection was performed independently in each dataset, and only predictors selected consistently were retained. This approach ensured that the final model captured robust associations, avoiding artifacts that could arise from any single imputation. This approach not only aligns with current methodological recommendations but also enhances the reproducibility and generalizability of our prediction model.

### Comparison with existing prediction models

Several pre-interventional tools have indeed been developed to predict functional outcomes after EVT. In a 2024 cohort study of 111 patients, the THRIVE, ASTRAL, and iScore scales achieved AUCs of 0.713, 0.738, and 0.820, respectively, for predicting prognosis after mechanical thrombectomy (38). In a large-scale analysis from the German Stroke Registry (*n* = 6,726), the PRE score yielded an AUC of 0.757 (95% CI: 0.747–0.768) and THRIVE-EVT achieved 0.751 (95% CI: 0.740–0.761) for predicting good functional outcome (39). A 2024 systematic review and meta-analysis reported that in studies predicting functional outcome after mechanical thrombectomy, the pooled test set AUC of machine learning models was 0.84 (95% CI: 0.79–0.88) (40).

Our model achieved a pooled test AUC of 0.795 (95% CI: 0.761–0.829), which falls within the range reported for contemporary pre-interventional tools and compares favorably with several established scores. However, direct comparisons should be interpreted with caution due to differences in study design, outcome definitions, population characteristics, and validation strategies. Notably, some models with higher reported AUCs incorporate postprocedural data (e.g., 24-h NIHSS), whereas our model was intentionally designed as a preoperative tool using only baseline variables to enable risk stratification before EVT (39). Thus, our model offers a pragmatic, multi-domain approach that integrates clinical, imaging, and laboratory markers without requiring post-procedural information. External validation in independent cohorts is needed to further evaluate its generalizability.

### Limitations

This study has several limitations. First, the model was developed and evaluated using a training/test split within a single-center dataset. While this provides some internal assessment of model performance, it does not fully address the question of generalizability. The absence of external validation in an independent cohort limits the ability to assess how well the model would perform in other settings or populations. External validation in multicenter cohorts is therefore needed to confirm the robustness and clinical applicability of our findings. Second, as a retrospective study, the results may be subject to unmeasured confounding factors, precluding definitive conclusions about causality. Additionally, follow up assessments were conducted primarily through telephone interviews, complemented by in person outpatient evaluations. While efficient, telephone follow up relies heavily on patients’ subjective reports, which could introduce recall bias. To mitigate this, dedicated follow up staff were trained, and a standardized follow up protocol was implemented. Regarding missing data, multiple imputation assumes data are missing at random, an assumption that may be violated in critically ill patients with missing laboratory tests. The imputation model also omitted certain outcome-related covariates, such as post-discharge interventions. For variable selection, the *p* < 0.2 screening threshold may overlook nonlinear effects, while requiring 100% selection frequency across imputed datasets, though ensuring robustness, may exclude clinically relevant predictors present in most but not all datasets.

## Conclusion

We developed a multidimensional model integrating clinical, imaging, and laboratory markers to predict futile reperfusion following EVT in patients with anterior circulation stroke. In practice, this model may help identify patients at particularly high risk for poor recovery, thereby supporting clinical decision making and postprocedural care planning. Notably, several predictors in the model, such as hyperglycemia and elevated inflammatory markers, are potentially modifiable. Whether targeted interventions addressing these factors can improve outcomes warrants further investigation.

## Data Availability

The original contributions presented in the study are included in the article/Supplementary material, further inquiries can be directed to the corresponding author.
