# Supplementary material for: Clinical multidimensional prediction model for futile reperfusion in acute ischemic stroke after endovascular thrombectomy
Source: Front Neurol. 2026 Apr 30;17:1819703. doi: 10.3389/fneur.2026.1819703 (PMC13171379; doi:10.3389/fneur.2026.1819703)
Supplement: Supplementary file 1 [file Supplementary_file_1.DOCX]

**Supplemental Figures**

**Supplemental Figure 1. Frequency of Variable Selection Across Five Multiply Imputed Datasets**

Bar plot showing the selection frequency of candidate predictors across five multiply imputed datasets. The final predictor set comprised nine variables selected in all five imputed datasets (frequency: 100%): NIHSS, CTA-SI ASPECTS, time from OTR, CCS, CRP, glucose, white blood cell count, neutrophil count, and monocyte count. Lymphocyte count was selected in four datasets (80%). Variables with selection frequency <100% were excluded from final model development.

Abbreviations: NIHSS, National Institutes of Health Stroke Scale; CTA-SI ASPECTS, Computed Tomography angiography-source images Alberta Stroke Program Early Computed Tomography Score; OTR, onset to reperfusion; CCS, collateral circulation scores; CRP, C-reactive protein; WBC, white blood cell.

**
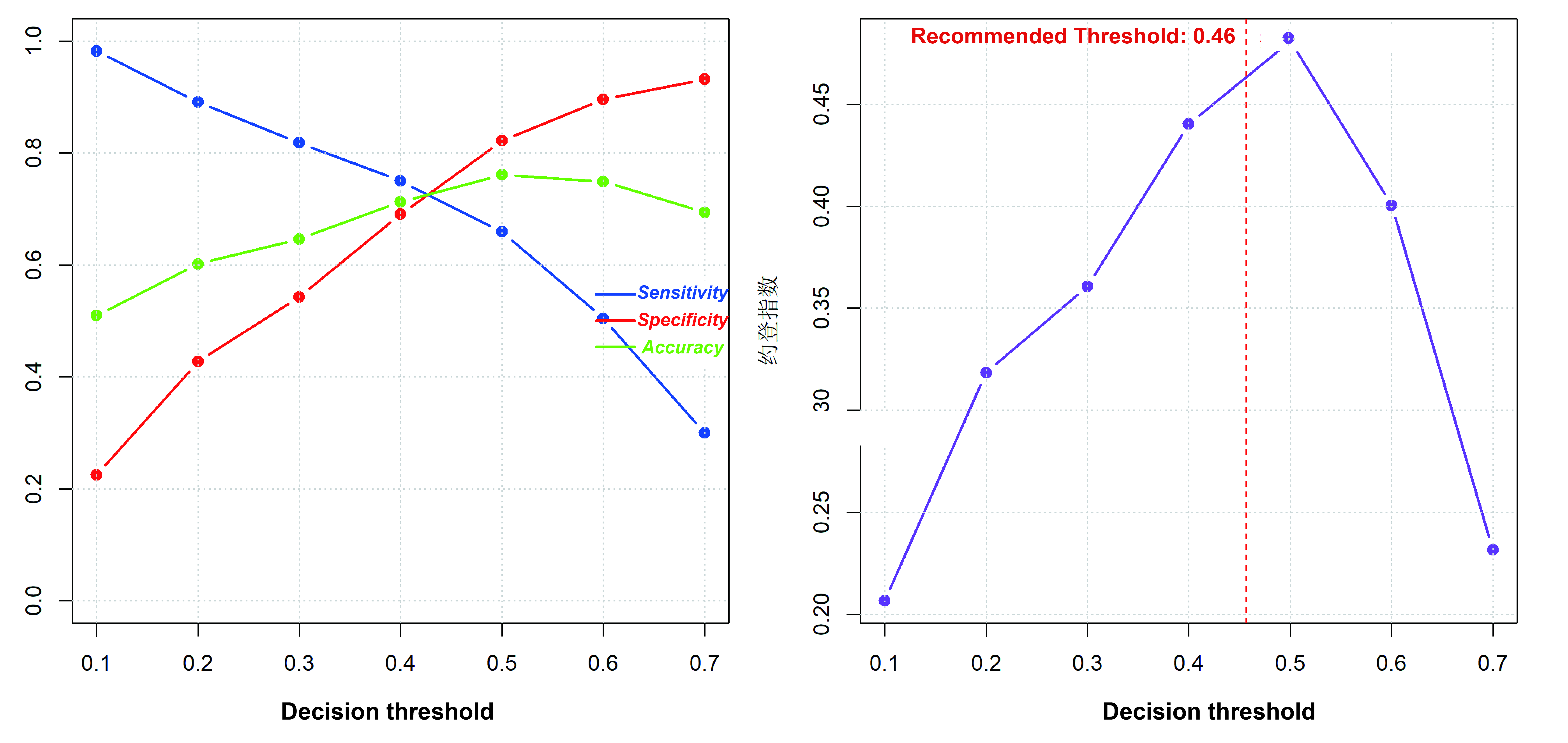
**

**Supplemental Figure 2. Optimal threshold selection based on sensitivity-specificity trade-off and Youden's index**

(Left)​Variation of sensitivity, specificity, and overall accuracy across decision thresholds from 0.1 to 0.7. Sensitivity decreases while specificity increases with higher thresholds. The overall accuracy peaks near the intersection of sensitivity and specificity. (Right)​Youden's index across thresholds, with maximum value indicating the optimal trade-off between sensitivity and specificity. The recommended decision threshold is 0.46.
